# Reliable and efficient magnetic data inversion for resource detection using a hybrid bat algorithm

**DOI:** 10.1038/s41598-025-10138-3

**Published:** 2025-07-26

**Authors:** Khalid S. Essa, Mahmoud Elhussein, Omar A. Gomaa, Zein E. Diab

**Affiliations:** Geophysics Department, Faculty of Science, Cairo University, Giza, 12613 Egypt

**Keywords:** Magnetic inversion, Fourth horizontal gradient, Bat algorithm, Subsurface imaging, Faro mine, Geophysical exploration, Geomagnetism, Geophysics, Mineralogy

## Abstract

Accurate estimation of subsurface parameters is a critical objective in geophysical exploration. This study introduces an innovative hybrid algorithm integrating the Bat Algorithm (BA) with the Fourth Horizontal Gradient (FHG) to optimize the estimation of geometric parameters (depth, amplitude coefficient, shape factor, source origin, and magnetization angle) from magnetic field data. The FHG enhances the resolution of magnetic anomalies by mitigating regional field effects, while the BA efficiently navigates the parameter space to accurately delineate subsurface structures modeled as simplified geometric. This approach prioritize geometric parameters to define the spatial configuration of magnetic sources, assuming constant petrophysical properties (e.g., magnetization intensity, susceptibility contrast) to simplify the inversion process. The algorithm’s robustness and effectiveness were extensively evaluated using synthetic magnetic datasets under both noise-free and with 10% Gaussian noise conditions. The findings demonstrate the method’s capability to achieve accurate parameter estimation even in noisy environments. The proposed approach was further assessed using real magnetic profile data acquired from the Faro Mine Complex in Yukon, Canada. The estimated subsurface parameters closely match with well data and prior studies, emphasizing the algorithm’s practical effectiveness. This integrated approach significantly advances the interpretation of magnetic datasets by improving both the accuracy and resolution of subsurface parameters estimation within the framework of idealized geometric models.

## Introduction

Magnetic surveys are necessities in geophysical exploration since they reveal significant information about the subsurface structures and material properties. These surveys are regularly employed in numerous applications, including geothermal inquiry processes, observing the environment, research into archaeological sites^1–4^, and geotechnical estimations^4^. They are also beneficial in detecting unexploded ordnance (UXO)^5,6^, oil and gas searching^7^, dike recognition^8,9^, underground metallic object identification, interior cavity visualization, landfill inquiries, basement’s depth appraisal^10–13^, and plutonic igneous rock intrusion research investigations^14,15^. Additionally, magnetic methods are regularly employed in prospecting for minerals and geotectonic examination^16,17^, demonstrating their capacity for adaptability along with significance in geophysical investigations.

For quantifying subsurface parameters in the interpretation of magnetic anomalies, an essential geometrical structure such as sphere-shaped, horizontal cylindrical objects, and vertical cylindrical objects are frequently employed^18–23^. A variety of graphical and mathematical methodologies have been invented throughout the years, including matching curves^24,^ nomograms^25^, characteristic points methods^26,27^, Werner, Euler, and Located Euler (LED) deconvolution^28–30^, moving average techniques, least-squares methods^31,32^, Fourier transforms^33,34^, local wave number methods^35,36^, tilt-angle approaches^37,38^, correlation techniques^39^, spectral analysis^40,41^, analytic signal^42^, and recent approaches that depend on the gradients to estimate the subsurface parameters^43–45^. Despite their extensive use, these methods frequently encounter significant limitations, including subjectivity, reliance on limited data points, sensitivity to noise, and susceptibility to nearby effects that can compromise accuracy. Additionally, many of these methodologies require initial model parameters derived from geological assumptions, that potentially leading to solutions trapped in local minima rather than the global optimum.

To overcome these challenges, metaheuristic algorithms have been introduced for magnetic data interpretation. These algorithms are designed to achieve globally optimal solutions with greater precision and efficiency. Popular metaheuristic techniques include simulated annealing (SA)^46^, genetic algorithms (GA)^47^, particle swarm optimization (PSO)^48–53^, neural networks (NN), differential evolution (DE)^54,^ ant colony optimization (ACO)^55^, cuckoo optimization algorithm^56^, and bat algorithm (BA)^57,58^. These algorithms are known for their adaptability and efficacy in solving complex optimization problems.

This study adopts the Bat Algorithm (BA) for analyzing magnetic data and estimating subsurface parameters with simple geometric shapes. The BA is recognized for its efficiency in handling complex problems due to: (1) it ensures rapid convergence by transitioning from exploration to exploitation early in the process^59^, (2) it functions effectively as both a global and local optimizer^60^, (3) it manages multi-modal problems with ease^61^, and (4) it iteratively updates parameters to preserve solution diversity^60^. BA has been successfully applied across diverse fields, including micro-smart grids, economic scheduling, fault diagnosis, multi-objective optimization, and echo state networks^62^.

In the current article, we incorporate the FHG with the BA to come up with the fourth horizontal gradient bat algorithm (FHGBA), which proposes a refined approach for magnetic data analysis and interpretation. The FHG, as a higher-order gradient, enhances magnetic data resolution by competently suppressing regional trends modeled by a third-order polynomial. The FHGBA approach provides extensive and precise investigation of the magnetic anomalies through using varied window sizes, allowing for more accurate detection and characterization of subsurface objects.

The core objective of the current article is to capitalize on the FHG and the BA to further advance the analysis of magnetic data and subsurface parameter estimation. The proposed FHGBA method is validated using synthetic models and real-world field data, demonstrating its ability to improve both the accuracy and reliability of geophysical interpretations.

## Methodology

In geophysical exploration, accurately interpreting magnetic data to estimate subsurface source parameters requires a robust inversion approach with advanced capabilities. This study employs a novel methodology that integrates the FHG with the BA to improve the precision and reliability of subsurface parameter estimation.

### Forward modeling

**Fig. 1:**
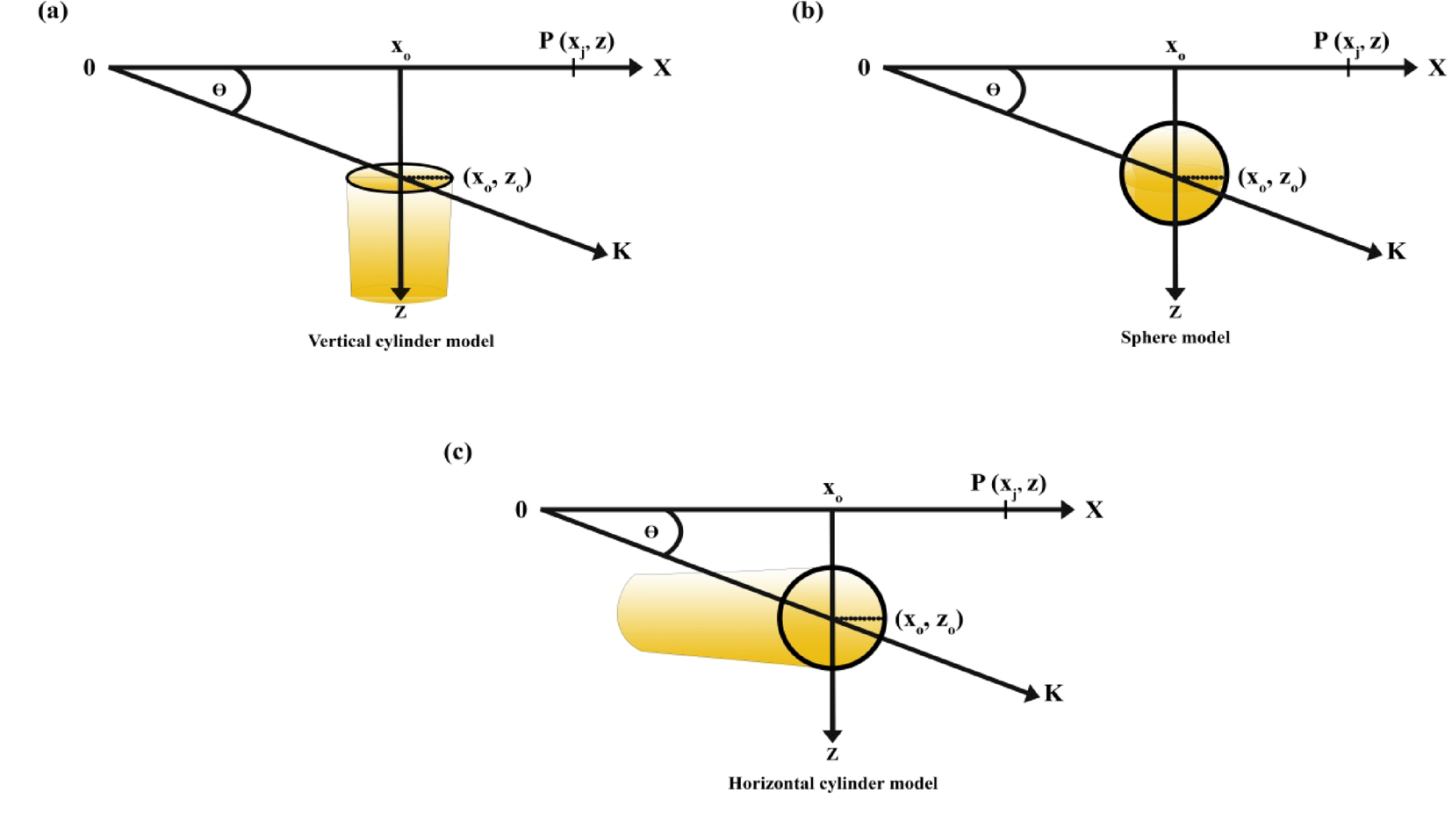
Visualization of subsurface geometric models. **a** Vertically oriented cylinder. **b** Spherical model. **c.** Horizontally oriented cylinder.

Forward modeling simulates magnetic anomalies generated by basic geometric shapes, such as spheres, cylinders, and thin sheets (Fig. 1). Mathematical expressions were applied to calculate synthetic anomalies, enabling realistic data simulation for validation purposes:

1 $$\:{T}_{obs}\left({x}_{j}\right)=\:{T}_{residual}\left({x}_{j},\:z,\:\theta,\:{x}_{o},K,q\right)+\:{T}_{regional}\left({x}_{j},\:{x}_{o}\right),\:j=\text{1,2},3,\:\dots\:,\:n\:$$

2 $$\:{T}_{residual}\left({x}_{j},\:z,\:\theta,\:{x}_{o},K,q\right)=\:K*\left(\frac{D{z}^{2}+F\left({x}_{j}-\:{x}_{o}\right)+G{\left({x}_{j}-\:{x}_{o}\right)}^{2}}{{\left({\left({x}_{j}-\:{x}_{o}\right)}^{2}+\:{z}^{2}\right)}^{q}}\right)\:,\:j=\text{1,2},3,\:\dots\:,\:n\:$$

3 $$\:{T}_{regional}\left({x}_{j},\:{x}_{o}\right)=\:{A}_{1}{\left({x}_{j}-\:{x}_{o}\right)}^{3}+{A}_{2}{\left({x}_{j}-\:{x}_{o}\right)}^{2}+{A}_{3}\left({x}_{j}-\:{x}_{o}\right)+{A}_{4}\:,\:j=\text{1,2},3,\:\dots\:,\:n\:$$

where $\:{x}_{j}$ and $\:{x}_{o}$ signify the measurement points and the origin of the source (in kilometers), z is the depth of the buried source (in kilometers), q is the shape factor describing the geometry of the body (dimensionless), K is the amplitude coefficient (in nT.$\:{km}^{2q-2}$), *θ* represents the magnetization angle (in degrees), $\:{T}_{obs}$ signify the observed magnetic data, $\:{T}_{residual}$ is the residual part of the magnetic data, D, F, and G are coefficients specific to each geometric shape (detailed in Table 1), and n is the number of data points along the profile. $\:{T}_{regional}$ is the regional part of the magnetic data modeled using a third degree polynomial function to approximate large scale regional trends in the observed magnetic data. $\:{A}_{1}$, $\:{A}_{2}$, $\:{A}_{3}$, and $\:{A}_{4}$ are the polynomial coefficients that describe the spatial variation of the regional magnetic data. This polynomial representation helps to isolate the long wavelength components associated with deep seated sources, allowing for improved resolution of residual anomalies.

**Table 1:** Terms used to simulate magnetic data for different geological models (sphere, cylinder, and thin sheet) under total, horizontal, and vertical fields. The table shows how the mathematical expressions vary based on the shape of the subsurface structure and the type of field being analyzed.

Model type	D	F	G	Field type
Sphere	$\:3\text{sin}2\left(\theta\right)-1$	$\:-3z\text{sin}\left(2\theta\right)$	$\:3\text{cos}2\left(\theta\right)-1$	Total
	$\:-\text{cos}\left(\theta\right)$	$\:-3z\text{sin}\left(\theta\right)$	$\:2\text{cos}\left(\theta\right)$	Horizontal
	$\:2\text{sin}\left(\theta\right)$	$\:-3z\text{cos}\left(\theta\right)$	$\:-\text{sin}\left(\theta\right)$	Vertical
Infinite horizontal cylinder, thin sheet (first horizontal derivative), and geological contact (second horizontal derivative)	$\:\text{cos}\left(\theta\right)$	$\:2z\text{sin}\left(\theta\right)$	$\:-\text{cos}\left(\theta\right)$	All fields
Thin sheet and geological contact (first horizontal derivative)	$\:\frac{\text{cos}\left(\theta\right)}{z}$	$\:\text{sin}\left(\theta\right)$	0	All fields

### Fourth horizontal gradient (FHG)

The FHG is a higher-order derivative technique designed to enhance magnetic data resolution and improve subsurface parameter estimation by suppressing regional field effects and amplifying localized anomaly features that are typically associated with near-surface sources. In contrast to raw magnetic anomalies, which are frequently influenced by long-wavelength regional fields that obscure subtle subsurface structures, the FHG (see Eq. 4) effectively isolates the short-wavelength component associated with shallow geological sources. Unlike lower-order gradients (e.g., second or third horizontal gradients), which are limited to removing first- or second-order polynomial trends^52,53,63^, the FHG is capable of suppressing more complex regional effects that can be represented by a third order polynomial model (Eq. 3). This higher-order suppression capability enhances the signal-to-noise ratio, making the FHG particularly suitable for delineating closely spaced or superimposed magnetic sources with greater clarity.

The FHG is computed using variable window length s, which adapts to different geological conditions. By applying the FHG operator to (Eq. 1), the gradient at an observation point x_j_​ is given as:

4 $$\:\varDelta\:{T}_{xxxx}\left({x}_{j},s\right)=\frac{T\left({x}_{j}+4s\right)-4T\left({x}_{j}+2s\right)+6T\left({x}_{j}\right)-4T\left({x}_{j}-2s\right)+T\left({x}_{j}-4s\right)}{16{s}^{4}}\:,$$

where s = 1, 2, 3, …, N represents window lengths and x_j_ is the observation point.

The data refined by the FHG method, using varying window lengths, is input into the BA for optimization. This combination significantly enhances subsurface parameter estimation by reducing noise and regional effects.

### Bat algorithm (BA)

The BA is a nature-inspired metaheuristic optimization approach constructed around the echolocation behavior of bats by Yang^59^, was applied to the interpretation of magnetic data. In darkness, bats utilize echolocation to locate their colony, avoid obstacles, and hunt prey. They emit intense sound pulses in the 8–10 kHz band and sense echoes reflected from nearby objects. Each pulse barely lasts only a few milliseconds (i.e., 8–10 ms). As bats draw closer to their target or prey, their pulse rate gets higher while their loudness drops^59^. This natural echolocation behavior can be mathematically formulated to optimize or maximize objective functions. The BA operates based on key principles: (1) Bats detect distances using sonar to locate obstacles and prey; (2) they fly within a specific frequency band [R_min_, R_max_] and characterized by a primary velocity (C_i_) at position (X_i_) to sense their environment; (3) their loudness (D_i_) and pulse emission rate (E_i_) adjust based on their proximity to the target.

**Fig. 2:**
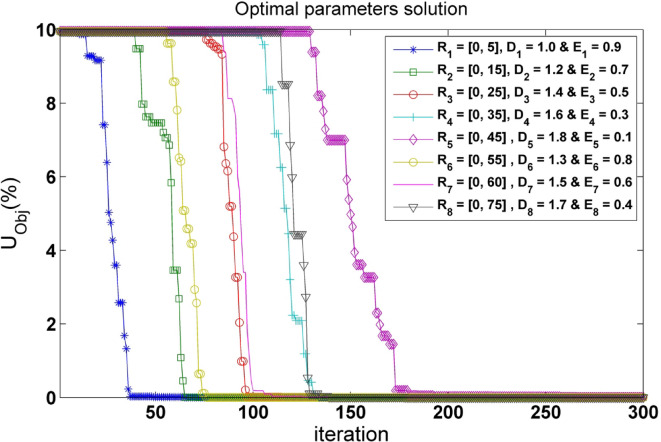
Convergence curves for optimal parameter selection. Displays the progression of parameter optimization in the algorithm, illustrating trends toward achieving accurate results.

The movement domain of the bats in the optimization problem may be altered by varying the frequency or wavelength. Therefore, choosing the right frequency is essential. Bats update their frequency (R_i_) in the optimization search space using the following equation:

5 $$\:{R}_{i}^{t}=\:{R}_{min}+\left({R}_{max}-\:{R}_{min}\right)*\:\beta\:$$

where, $\:{R}_{i}^{t}$ is the frequency of the *i*^th^ bat at iteration (*t*), $\:{R}_{max}$ and $\:{R}_{min}$ construct the permitted frequency band, and $\:\beta\:$ is a uniformly distributed random variable within [0, 1]. It needs to be selected such that it complements the size of the interest zone before being toned down to lower limits. After utilizing the approach with various settings, the optimal frequency band for the inquiry was found to be in the spectrum of [0, 5 Hz] (Fig. 2).

For each iteration, the velocity (C_i_) and position (X_i_) of bats were updated using the following two equations:

6 $$\:{C}_{i}^{(t+1)}=\:{C}_{i}^{t}+\left({X}_{i}^{t}-\:{X}_{best}\right)*\:{R}_{i}^{t}\:$$

7 $$\:{X}_{i}^{(t+1)}=\:{X}_{i}^{t}+{C}_{i}^{(t+1)}\:\:$$

where, $\:{C}_{i}^{t}$ is the velocity of the *i*^th^ bat at iteration *t*, $\:{X}_{best}$ signifies the best global solution found so far, and $\:{X}_{i}^{t}$ denotes the current position of the *i*^th^ bat. These equations explain bat movement strategies in the optimization process. The velocity update (Eq. 6) incorporates the consequence of the best global solution, that leads bats to optimal positions while conserving search space various perspectives. The position update (Eq. 7) incorporates the changed velocity, facilitating ongoing adaptation to superior solutions.

As bats draw closer to an optimal solution, their loudness (D_i_) and pulse emission rate (E_i_) fluctuate interactively to refine the search. The equations that regulate these adjustments are as follows:

8 $$\:{D}_{i}^{(t+1)}=\:\alpha\:{D}_{i}^{t}\:$$

9 $$\:{E}_{i}^{t}=\:{E}_{i}^{o}\left[1-exp(-\gamma\:\tau\:)\right]\:$$

where, $\:\alpha\:$ is a constant in the band 0 < $\:\alpha\:$ < 1 controlling loudness reduction, $\:{D}_{i}^{t}$ and $\:{E}_{i}^{t}$ are the loudness and the pulse emission rate of the *i*^th^ bat at iteration *t*, $\:\gamma\:$ is a positive constant regulating the rate of pulse emission increase, $\:\tau\:$ is a scaling factor, and $\:{E}_{i}^{o}$ signifies the initial pulse emission rate. The range of the pulse rate E_i_ is [0–1], with 0 meaning no pulse emission and 1 signifying the greatest emission rate of the pulses. Additionally, D_i_ may frequently be in the [1, 2] range for the initial loudness^59^. In execution, as a bat comes up to an optimal solution, its loudness falls which restricts its exploration scope, whereas its pulse emission rate grows which demonstrates a stronger local search around better solutions^64,65^.

When a new best solution is discovered, BA integrates a local random search process to enhance the solution even further. The new solution is generated in the following manner:

10 $$\:{X}_{new}=\:{X}_{old}+\:\epsilon\:{D}^{t}\:$$

where, $\:{D}^{t}$ defines the average loudness of all bats at iteration *t*, $\:\epsilon\:$ is a randomly generated number within [−1, 1]. This local exploitation mechanism allows BA to fine tune solutions by making small perturbations around the best solutions, increasing its ability to escape local minima.

The convergence behavior of BA process depends on a proper selection of parameters such as pulse emission rate (E_i_), frequency (R_i_), and loudness (D_i_). Through experimentation, the optimal configuration was determined according to Fig. 2, the best set is one with (R_1_= [0, 5], D_1_ = 1, and E_1_ = 0.9), because it delivers speedy convergence to the optimal solution and has the least NRMSE of the objective function in comparison to other sets. BA also shares commonalities with other optimization approaches. If the frequency fluctuations are substituted by a random parameter, and both (D_i_) and (E_i_) are assigned to one, the BA ultimately resembles the ordinary particle swarm optimization (PSO) algorithm^59^. This demonstrates the capability for adaptation to various optimization conditions.

### Inversion process

A novel code named “Inversion FHGBA” has been developed to provide an effective solution for identifying the optimal subsurface sources that align with real data. This code emphasizes key parameters such as depth, location, body shape, amplitude coefficient, and magnetization angle (denoted as z, $\:{x}_{o}$, q, K, *θ*) to achieve the best solution. The iterative process involves bats navigating randomly through the search space, refining their solutions, and identifying the most suitable positions based on performance evaluations. The solution with the lowest misfit function value is considered the optimal position, referred to as $\:{X}_{best}$. During each iteration, solutions are compared, and the best candidate is selected. After completing a predefined number of iterations, the final optimal solution is determined as $\:{X}_{best}$. To validate its accuracy and reliability, the FHGBA inversion code has been extensively tested on synthetic models before being applied to real-world datasets.

**Fig. 3:**
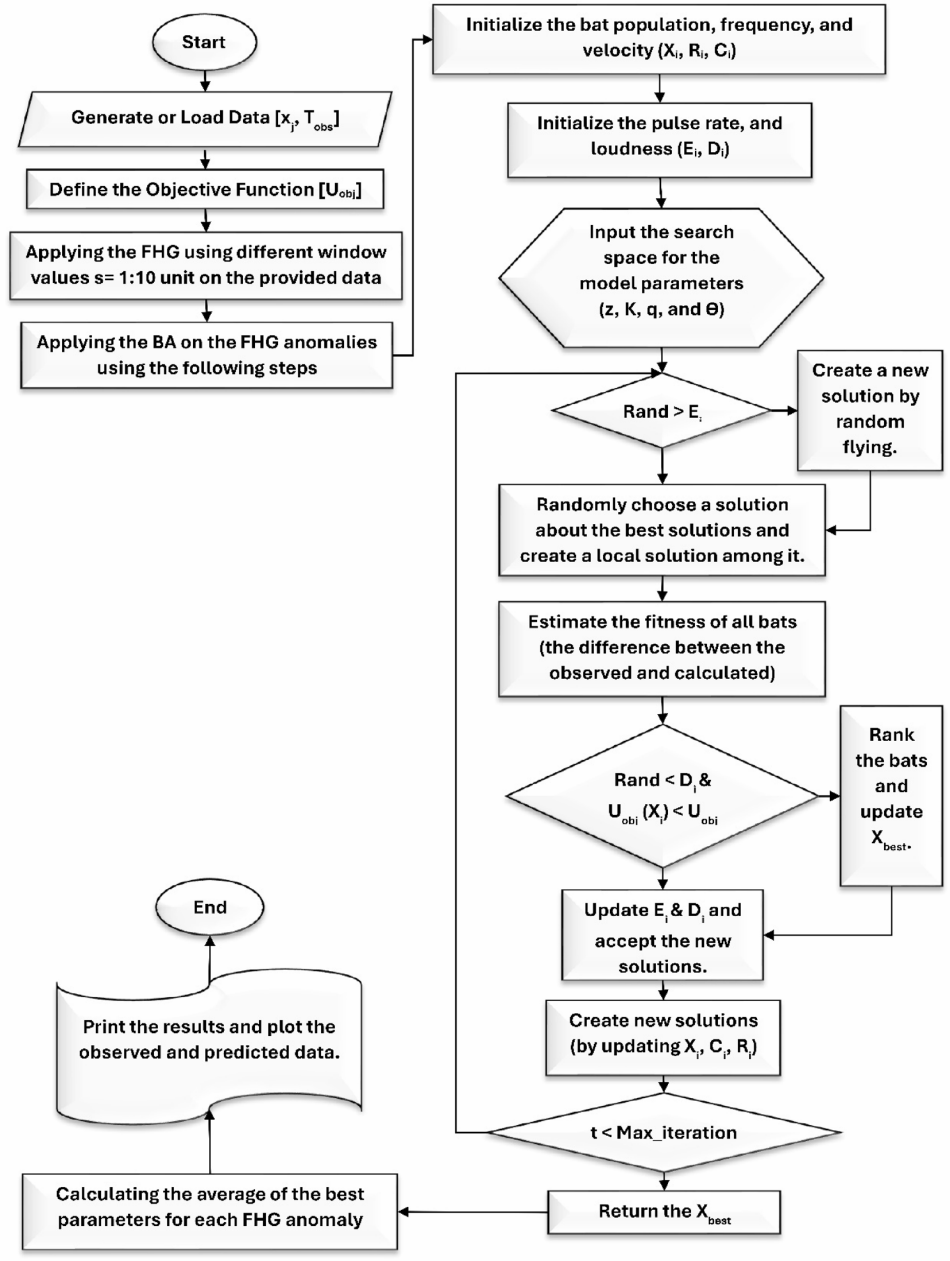
Application Flowchart of the Bat Algorithm on Fourth Horizontal Gradient Anomalies. Highlights the critical steps for parameter estimation and subsurface characterization.

The FHGBA method integrates the FHG using varying window sizes. The process begins by creating objective functions from magnetic data. FHG is then carefully applied within different window ranges to ensure consistency across anomalies. The BA is employed to optimize each gradient anomaly individually. The procedural flow of the algorithm is illustrated in Fig. 3. This multi-windows averaging approach overcomes the limitations associated with single window length, which may be sensitive to specific geological conditions. By avoiding reliance on a single window length, the method enhances the general applicability and robustness of subsurface structural interpretations. Following the optimization of gradient anomalies, results are aggregated and averaged to enhance robustness, significantly reducing uncertainties and errors in parameter estimation. By combining the intelligence of bat-inspired optimization with statistical averaging, the algorithm achieves greater accuracy in detecting subsurface features. This enhanced reliability makes the estimated parameters highly valuable for geophysical exploration.

The objective function ($\:{U}_{Obj}$) employed in this code calculates the normalized root-mean-square error (NRMSE) between observed and modeled magnetic anomalies. It is defined as:

11 $$\:{\text{U}}_{\text{O}\text{b}\text{j}}=\:\frac{100}{\text{M}\text{a}\text{x}\left({\text{T}}_{\text{o}\text{b}\text{s}}\right)-\text{M}\text{i}\text{n}\left({\text{T}}_{\text{o}\text{b}\text{s}}\right)}\:\sqrt{\frac{\sum_{\text{j}=1}^{\text{N}}{\left[{\text{T}}_{\text{o}\text{b}\text{s}}-\:{\text{T}}_{\text{c}\text{a}\text{l}}\right]}^{2}}{\text{N}}}\:\:\:$$

where N is the number of data points, $\:{\text{T}}_{\text{o}\text{b}\text{s}}$ represents the observed magnetic, and $\:{\text{T}}_{\text{c}\text{a}\text{l}}\:$signifies the calculated magnetic. Initially, (Eq. 11) is utilized to calculate misfits, and then the bat with the least misfit is selected as $\:{X}_{best}$.

## Results and discussion

### Synthetic datasets

The FHGBA was rigorously evaluated under diverse and challenging scenarios to determine its reliability and effectiveness in magnetic data inversion. The assessment included the creation of synthetic datasets and the simulation of real-world complexities to analyze its performance comprehensively.

### Model 1: impact of noise levels

**Fig. 4:**
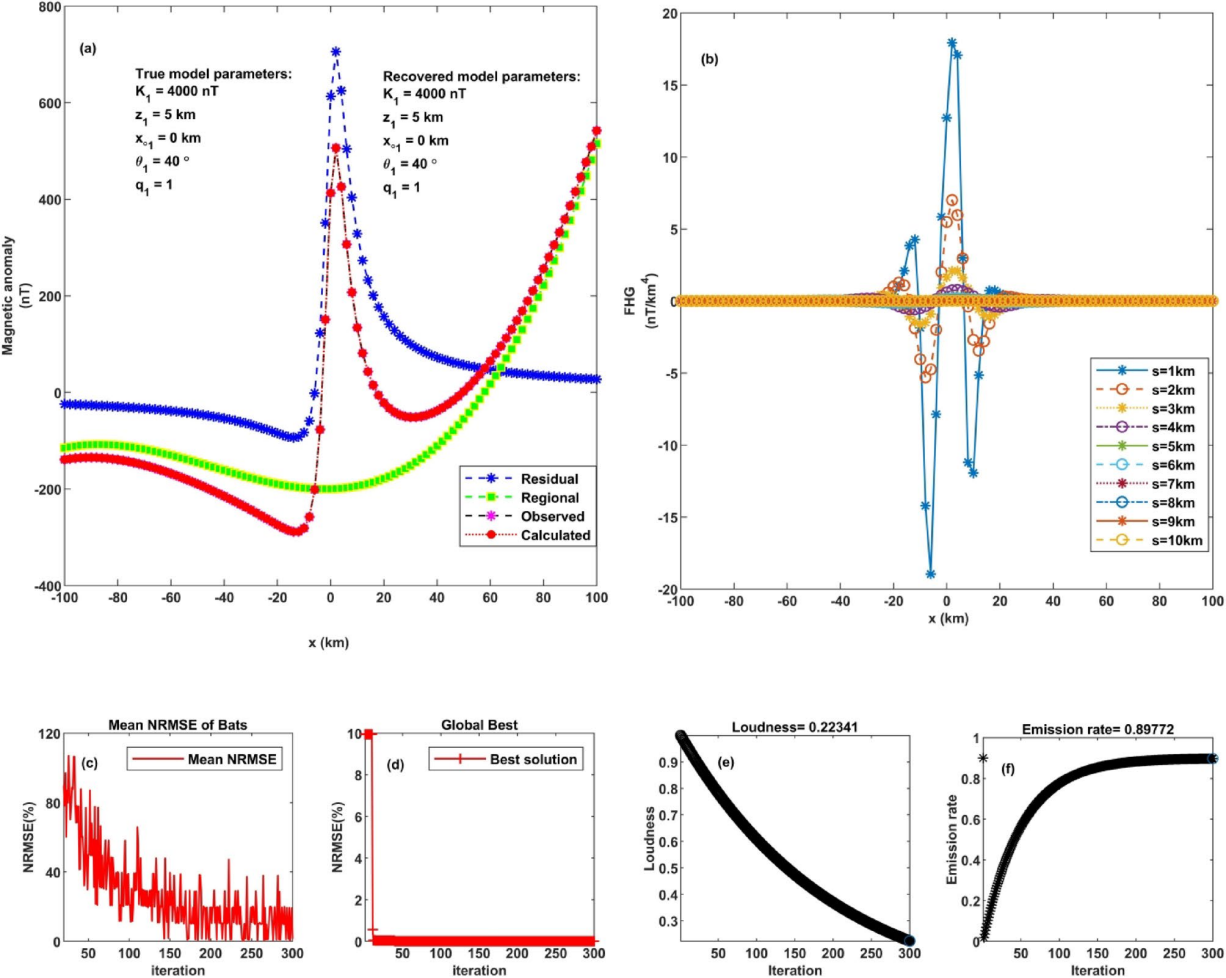
Noise-free thin sheet analysis. **a** Observed magnetic anomaly and computed optimal magnetic response using FHGBA. **b** FHG anomalies for varying ‘s’ window values. **c** Average NRMSE of all bats. **d** NRMSE of the global optimum solution ($\:{\varvec{U}}_{\varvec{O}\varvec{b}\varvec{j}}$) relative to iterations. **e** Loudness variation of bats. **f** Emission rate of bats.

This study explores the effectiveness of the FHGBA scheme in recovering source parameters from synthetic magnetic anomaly data. A theoretical magnetic anomaly was first generated using specific parameters (K = 140 nT, z = 5 km, q = 1.0, $\:{x}_{o}$ = 0 km, and *θ* = 40˚) over a 201-kilometer profile using Eq. (1) (Fig. 4a). This dataset served as the baseline for testing. The FHGBA approach was then applied to optimize the inversion process and accurately represent the magnetic response by analyzing FHG across different parameter values (s = 1, 2, 3, 4, 5, 6, 7, 8, 9, 10 km) (Fig. 4b). The optimization process aimed to minimize the normalized root-mean-square error (NRMSE) of the objective function. Figure 4c depicts the average NRMSE per iteration, demonstrating a consistent trend of convergence toward the optimal solution. This pattern reflects the algorithm’s stable performance and effective search behavior. Additionally, Fig. 4d shows the progression of the minimum NRMSE achieved by the individual components of the FHGBA–referred to as “microbats”. The results indicate that convergence is typically attained within 10 iterations for all microbats, confirming the method’s efficiency and rapid optimization capability in minimizing error during subsurface parameter estimation. Figure 4e and f illustrate the variation in average loudness and emission rate as modelled through the bat algorithm. The reduction in NRMSE highlights the FHGBA’s effectiveness in accurately retrieving the original parameters, supported by the evaluation of the generated magnetic data.

Table 2 summarizes the average estimated parameters derived from various FHG anomalies. The parameters are as follows: K = 4000 ± 6.67 nT, z = 5 ± 0.08 km, $\:{x}_{o}$= 0 ± 0.82 km, q = 1 ± 0.04, *θ* = 40˚ ± 0.82, these values characterize a horizontal cylinder. The calculated NRMSE was 4.12 × 10^−5^.

**Table 2:** Estimation of subsurface parameters for the noise-free synthetic magnetic field model (thin sheet/dike or first horizontal derivative of geological contact). The table presents the true model parameters, search ranges, and estimated values for different window sizes of the fourth horizontal gradient (s = 1 to s = 10 km). It also shows the average parameter estimates with uncertainties and the best objective function value.

Model parameters	True model parameters	Search range	Estimated parameters										Avg. value ± Uncertainty	$\:{\varvec{U}}_{\varvec{O}\varvec{b}\varvec{j}}$
			s = 1 km	s = 2 km	s = 3 km	s = 4 km	s = 5 km	s = 6 km	s = 7 km	s = 8 km	s = 9 km	s = 10 km		
K (nT)	4000	3000–4500	4010	4000	4000	3990	4010	4000	3990	4000	4000	4000	4000 ± 6.67	4.12 × 10^−5^
z (km)	5	1–10	4.9	4.9	4.9	5.1	5	5.1	5	5	5	5.1	5 ± 0.082	
$\:{\varvec{x}}_{\varvec{o}}$(km)	0	−10–10	−1	1	0	1	0	−1	0	−1	0	1	0 ± 0.82	
q	1	0–2.5	0.95	0.95	1	1	1.05	1.05	1.05	1	1	0.95	1 ± 0.04	
*θ*	40	0–90	40	39	39	41	40	40	39	41	40	41	1 ± 0.81	

To further evaluate the robustness of the FHGBA, gaussian random noise with a mean of zero and a standard deviation of one nT was introduced into synthetic data at a 10% level. The noise level was calculated using the following equation [23]:

12 $$Noise~level~\left( \% \right)=~\frac{{\left\| {{T_{noisy}} - T} \right\|}}{{\left\| {{T_{noisy}}} \right\|}}~ \times 100~\%$$

where, $\:T$ and $\:{T}_{noisy}\:$represent the noise-free and noisy magnetic data values.

**Fig. 5:**
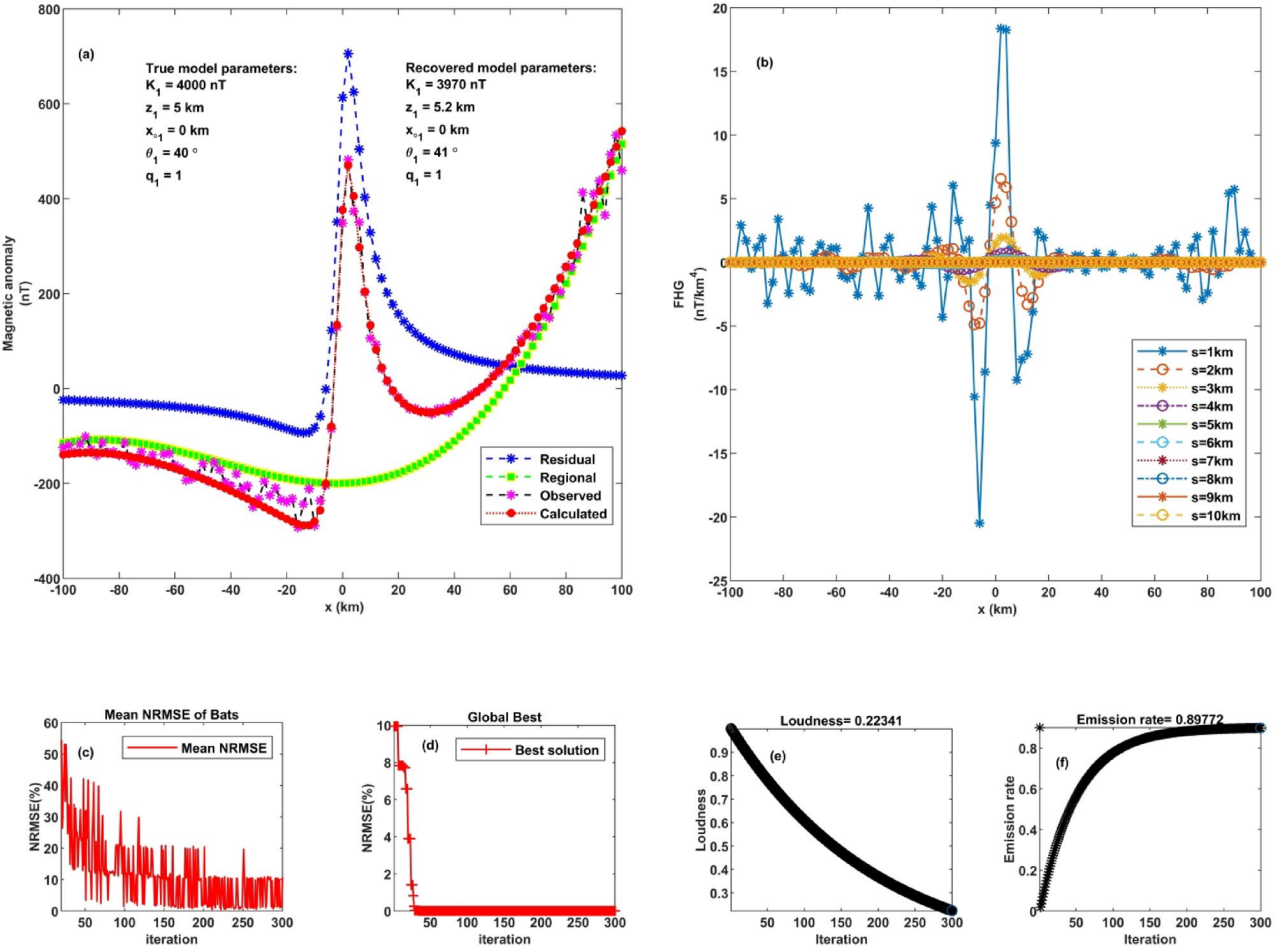
Thin sheet under 10% noise. **a** Observed magnetic anomaly and computed optimal magnetic response using FHGBA. **b** FHG anomalies for varying ‘s’ window values. **c** Average NRMSE of all bats. **d** NRMSE of the global optimum solution ($\:{\varvec{U}}_{\varvec{O}\varvec{b}\varvec{j}}$) relative to iterations. **e** Loudness variation of bats. **f** Emission rate of bats.

**Fig. 6:**
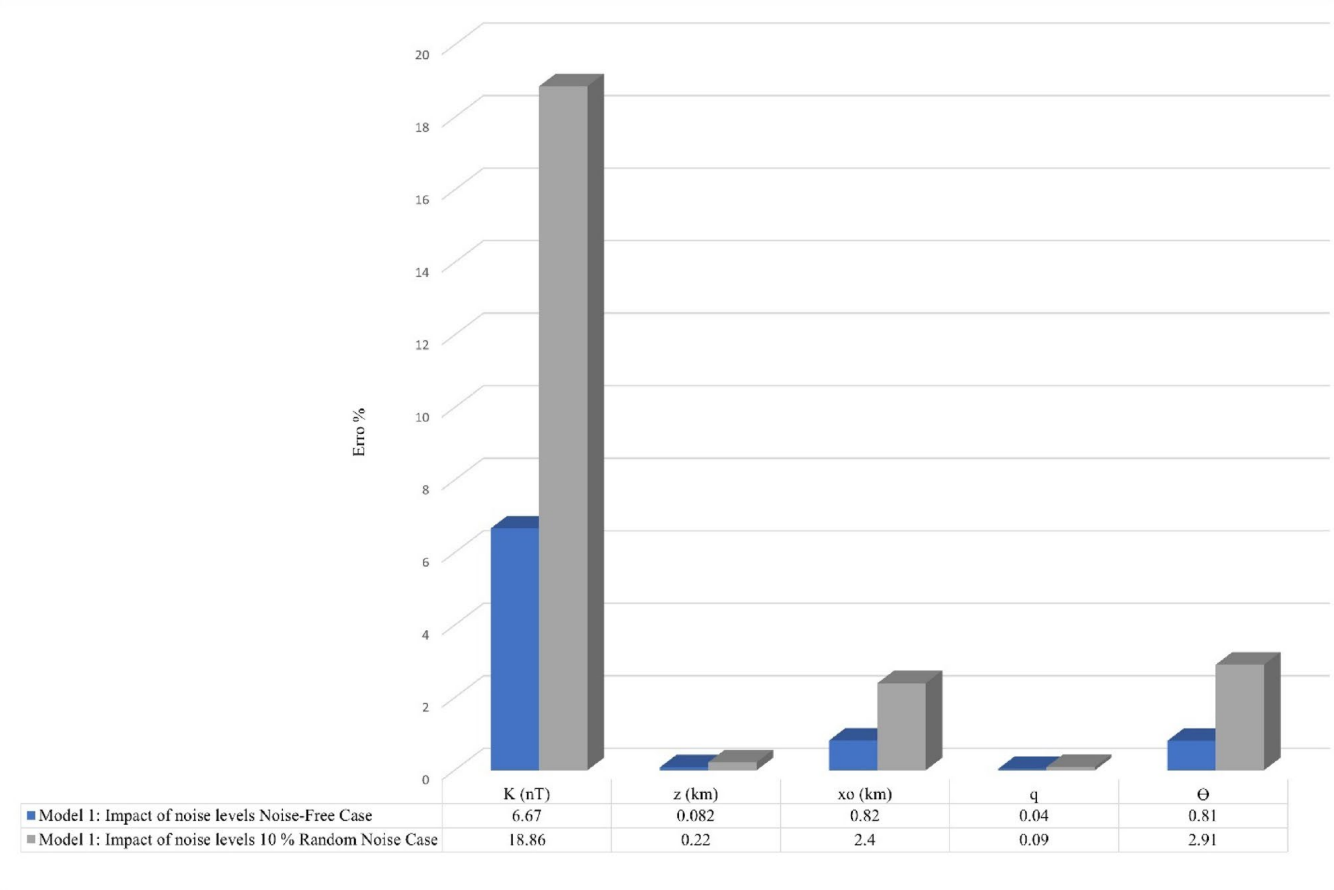
The relative errors of each parameter demonstrate the accuracy and the robustness of our algorithm across varying noise levels, highlighting the ability to maintain reliable and stable performance in the presence of significant noise levels.

Figure 5 demonstrates the algorithm’s performance under these conditions. Despite the added noise, the FHGBA successfully identified optimal model parameters by minimizing the NRMSE (Fig. 5a). The estimated parameters closely matched the original values. Figure 5b illustrates the FHG anomalies for the noisy data, while (Fig. 5c and d) depict the mean NRMSE values and the convergence process, respectively. Figure 5e and f illustrate the variation in average loudness and emission rate as modelled through the bat algorithm. Figure 6 shows the relative errors to each parameter, which demonstrates the accuracy and the robustness of our algorithm across varying noise levels, highlighting the ability to maintain reliable and stable performance in the presence of significant noise levels.

Table 3 presents the estimated parameters for the noise-affected model. The estimated parameters are as follows: K = 3970 ± 18.86 nT, z = 5.2 ± 0.22 km, $\:{x}_{o}$= 0 ± 2.4 km, q = 1 ± 0.09, *θ* = 41˚ ± 2.91. The NRMSE for this scenario was calculated as 0.0002.

**Table 3:** Estimation of subsurface parameters for the synthetic magnetic field model with 10% noise (thin sheet/dike or first horizontal derivative of geological contact). The table presents the true model parameters, search ranges, and estimated values across different fourth horizontal gradient windows (s = 1 to s = 10 km). It includes the average parameter estimates with uncertainties and the best objective function value, showing the algorithm’s resilience to noise.

Model parameters	True model parameters	Search range	Estimated parameters										Avg. value ± Uncertainty	$\:{\varvec{U}}_{\varvec{O}\varvec{b}\varvec{j}}$
			s = 1 km	s = 2 km	s = 3 km	s = 4 km	s = 5 km	s = 6 km	s = 7 km	s = 8 km	s = 9 km	s = 10 km		
K (nT)	4000	3000–4500	3990	4010	3960	3980	3960	3950	3970	3970	3950	3960	3970 ± 18.86	0.000192
z (km)	5	1–10	5.3	5.5	4.9	5.1	5.2	5.1	5.1	5.4	5.5	4.9	5.2 ± 0.22	
$\:{\varvec{x}}_{\varvec{o}}$(km)	0	−10–10	0	−2	4	1	2	−1	−2	−3	3	−2	0 ± 2.4	
q	1	0–2.5	1.05	1.1	0.9	0.9	1	1.1	0.9	0.9	1.1	1.05	1 ± 0.09	
*θ*	40	0–90	44	42	38	38	43	44	37	38	42	44	41 ± 2.91	

The findings highlight the effectiveness of the FHGBA technique, showcasing its ability to reliably estimate model parameters even in the presence of noise. This validation underscores its applicability and robustness in geophysical exploration.

### Model 2: impact of regional anomaly

**Fig. 7:**
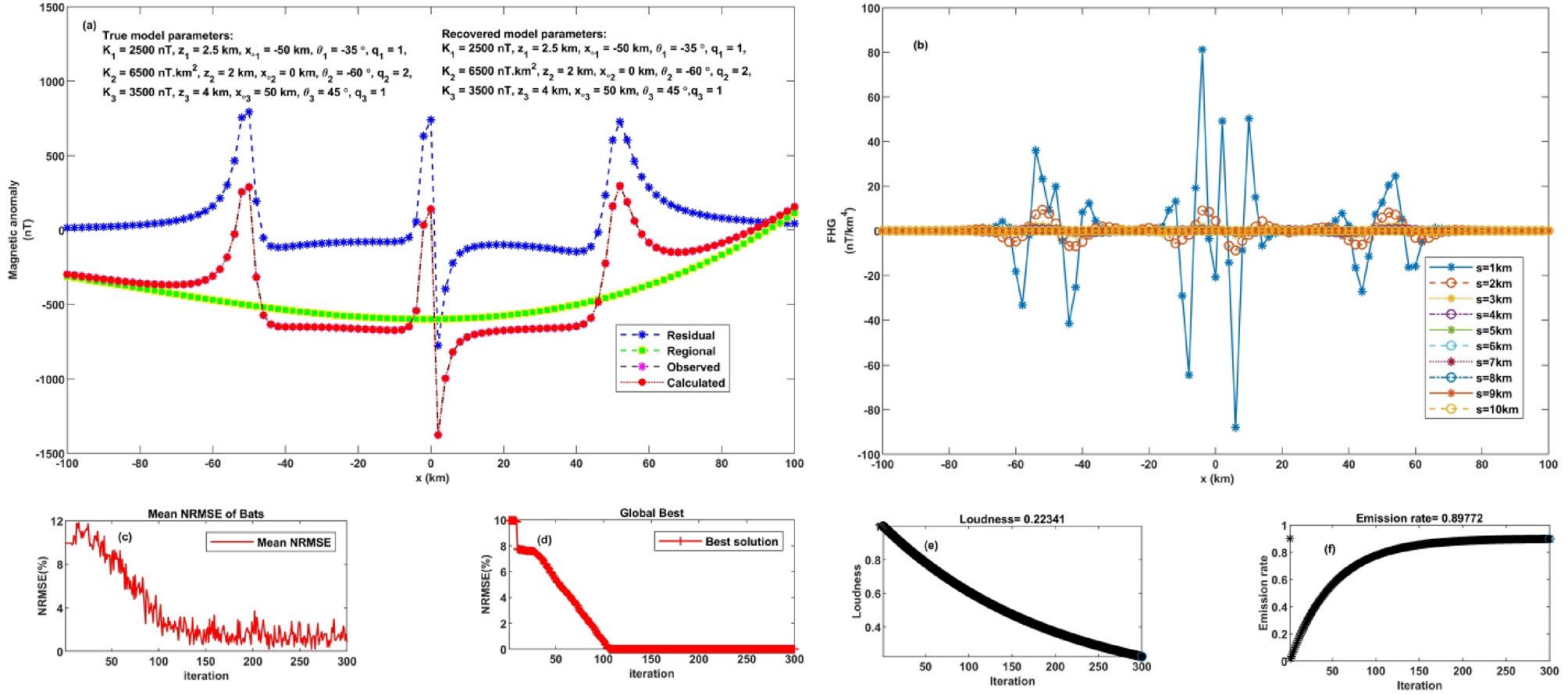
Noise-free multi-feature geological model analysis. **a** Observed anomaly and computed optimal response using FHGBA. **b** FHG anomalies for varying ‘s’ values. **c** Average NRMSE of bats. **d** NRMSE of global optimum ($\:{\varvec{U}}_{\varvec{O}\varvec{b}\varvec{j}}$) relative to iterations. **e** Loudness variation. **f** Emission rate of bats.

**Fig. 8:**
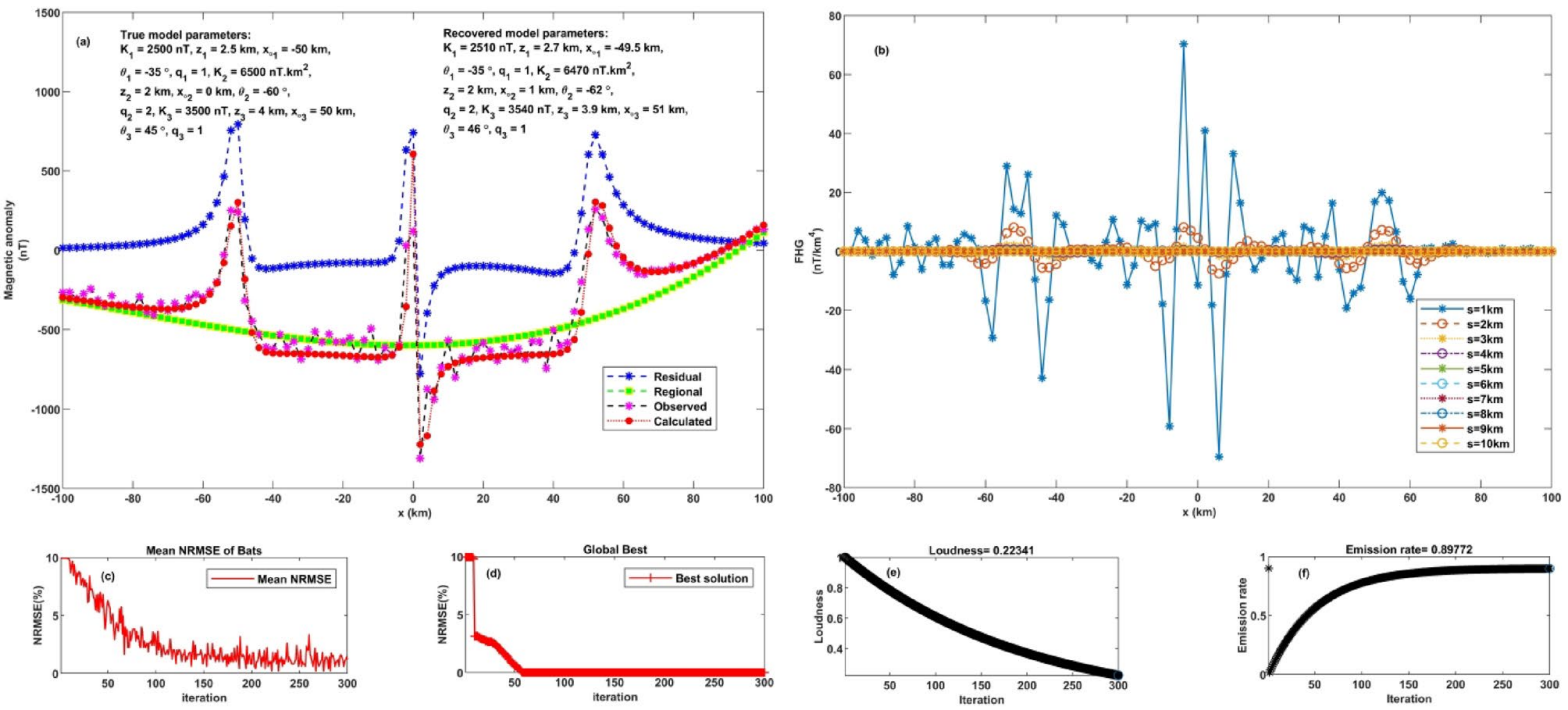
Multi-feature geological model analysis under 10% noise. **a** Observed anomaly and computed optimal response using FHGBA. **b** FHG anomalies for varying ‘s’ values, **c** Average NRMSE of bats. **d** NRMSE of global optimum ($\:{\varvec{U}}_{\varvec{O}\varvec{b}\varvec{j}}$) relative to iterations. **e** Loudness variation. **f** Emission rate of bats.

This section examines the FHGBA’s performance under more complex conditions, incorporating both regional anomalies and noise. The test involved generating a synthetic dataset by combining three subsurface models and adding a linear regional anomaly with random noise at a 10% level. A numerical model with specific parameters ($\:{K}_{1}$ = 2500 nT, $\:{z}_{1}$ = 2.5 km, $\:{q}_{1}$= 1, $\:{x}_{o1}$ = −50 km, and $\:{\theta}_{1}$ = −35˚) characterize a thin sheet, a horizontal cylinder body with the following parameters ($\:{K}_{2}$ = 6500 nT.km^2^, $\:{z}_{2}$ = 2 km, $\:{q}_{2}$= 2, $\:{x}_{o2}$ = 0 km, and $\:{\theta}_{2}$ = −60˚), and a thin dike with ($\:{K}_{3}$ = 3500 nT, $\:{z}_{3}$ = 4 km, $\:{q}_{3}$= 1, $\:{x}_{o3}$ = 50 km, and $\:{\theta}_{3}$ = 45˚) calculated using (Eq. 1), incorporating a linear regional trend $\:\left(0.0003*\:{\left({x}_{j}-\:{x}_{o}\right)}^{3}+0.04*\:{\left({x}_{j}-\:{x}_{o}\right)}^{2}+0.15*\:\left({x}_{j}-\:{x}_{o}\right)-200\right)\:$ and superimposed with 10% random noise (Figs. 7 and 8).

The FHGBA was then applied to estimate the original parameters by analyzing FHG anomalies across various window values (s = 1 to 10 km, Fig. 7b). The results demonstrating the algorithm’s effectiveness, recovering parameters close to the original values ($\:{K}_{1}$ = 2500 ± 9.43 nT, $\:{z}_{1}$ = 2.5 ± 0.08 km, $\:{q}_{1}$= 1 ± 0.08, $\:{x}_{o1}$ = −50 ± 0.82 km, and $\:{\theta}_{1}$ = −35˚ ± 0.81), ($\:{K}_{2}$ = 6500 ± 11.55 nT.km^2^, $\:{z}_{2}$ = 2 ± 0.19 km, $\:{q}_{2}$= 2 ± 0.14, $\:{x}_{o2}$ = 0 ± 2.67 km, and $\:{\theta}_{2}$ = −60˚ ± 2.94), and ($\:{K}_{3}$ = 3500 ± 14.91 nT, $\:{z}_{3}$ = 4 ± 0.36 km, $\:{q}_{3}$= 1 ± 0.06, $\:{x}_{o3}$ = 50 ± 4.64 km, and $\:{\theta}_{3}$ = 45˚ ± 2.06). The calculated NRMSE was 4.06 × 10^−6^ (Table 4), reflecting a good fit between the calculated and synthetic anomalies (Fig. 7a). Figure 7d illustrates the optimization process, showing the gradual reduction of the minimum NRMSE over approximately 100 iterations. Figure 7c further highlights the convergence through the average NRMSE per iteration. Figure 7e and f illustrate the variation in average loudness and emission rate as modelled through the bat algorithm.

**Table 4:** Results of subsurface parameter Estimation for a Noise-Free synthetic magnetic model with multiple geological features. This table presents the findings from the second synthetic magnetic example, which includes a thin sheet (dike), a horizontal cylinder, and an additional thin sheet (dike), along with a third-order regional field defined by (0.0003 ($\:{x}_{j}$ -$\:\:{x}_{o}$)^3^ + 0.04 ($\:{x}_{j}$ -$\:\:{x}_{o}$)^2^ + 0.15 ($\:{x}_{j}$ -$\:\:{x}_{o}$) − 200). It provides true model parameters, search ranges, estimated values for varying window sizes of the fourth horizontal gradient (s = 1 to s = 10 km), and the average estimates with associated uncertainties.

Model parameters	True model parameters	Search range	Estimated parameters										Avg. value ± Uncertainty	$\:{\varvec{U}}_{\varvec{O}\varvec{b}\varvec{j}}$
			s = 1 km	s = 2 km	s = 3 km	s = 4 km	s = 5 km	s = 6 km	s = 7 km	s = 8 km	s = 9 km	s = 10 km		
$\:{\varvec{K}}_{1}$(nT)	2500	2000–3500	2490	2490	2490	2510	2490	2510	2500	2500	2510	2510	2500 ± 9.43	4.06 × 10^−6^
$\:{\varvec{z}}_{1}$(km)	2.5	1–10	2.4	2.6	2.5	2.5	2.4	2.6	2.6	2.4	2.5	2.5	2.5 ± 0.08	
$\:{\varvec{x}}_{\varvec{o}1}$(km)	−50	−60 to −40	−50	−50	−50	−49	−49	−51	−51	−49	−51	−50	−50 ± 0.82	
$\:{\varvec{q}}_{1}$	1	0–2.5	1.05	0.9	1.1	1.1	0.9	0.95	1.05	1.05	0.95	0.95	1 ± 0.08	
$\:{\theta}_{1}$	−35	−90–90	−34	−34	−35	−36	−35	−34	−36	−36	−35	−35	−35 ± 0.81	
$\:{\varvec{K}}_{2}$(nT.km^2^)	6500	6000–7500	6520	6510	6490	6500	6480	6490	6500	6500	6510	6500	6500 ± 11.55	
$\:{\varvec{z}}_{2}$(km)	2	1–10	2.2	1.9	1.8	2	2.3	2.1	2.2	1.9	1.8	1.8	2 ± 0.19	
$\:{\varvec{x}}_{\varvec{o}2}$(km)	0	−10–10	−3	−4	−2	0	−2	2	1	3	4	1	0 ± 2.67	
$\:{\varvec{q}}_{2}$	2	0–2.5	1.9	2	2.2	2.1	1.9	1.9	1.8	2.1	2.2	1.9	2 ± 0.14	
$\:{\theta}_{2}$	−60	−90–90	−63	−59	−58	−59	−63	−62	−64	−57	−55	−60	−60 ± 2.94	
$\:{\varvec{K}}_{3}$(nT)	3500	3000–4500	3500	3530	3480	3520	3490	3490	3490	3500	3500	3500	3500 ± 14.91	
$\:{\varvec{z}}_{3}$(km)	4	1–10	4.2	4.4	4.4	4	3.5	3.9	3.3	4	4.2	4.1	4 ± 0.36	
$\:{\varvec{x}}_{\varvec{o}3}$(km)	50	−10–10	43	42	51	51	50	47	52	55	54	55	50 ± 4.64	
$\:{\varvec{q}}_{3}$	1	0–2.5	0.9	1	1	1	1.05	0.95	1.05	0.95	1	1.1	1 ± 0.06	
$\:{\theta}_{3}$	45	0–90	46	47	47	46	45	43	43	45	47	41	45 ± 2.06	

**Fig. 9:**
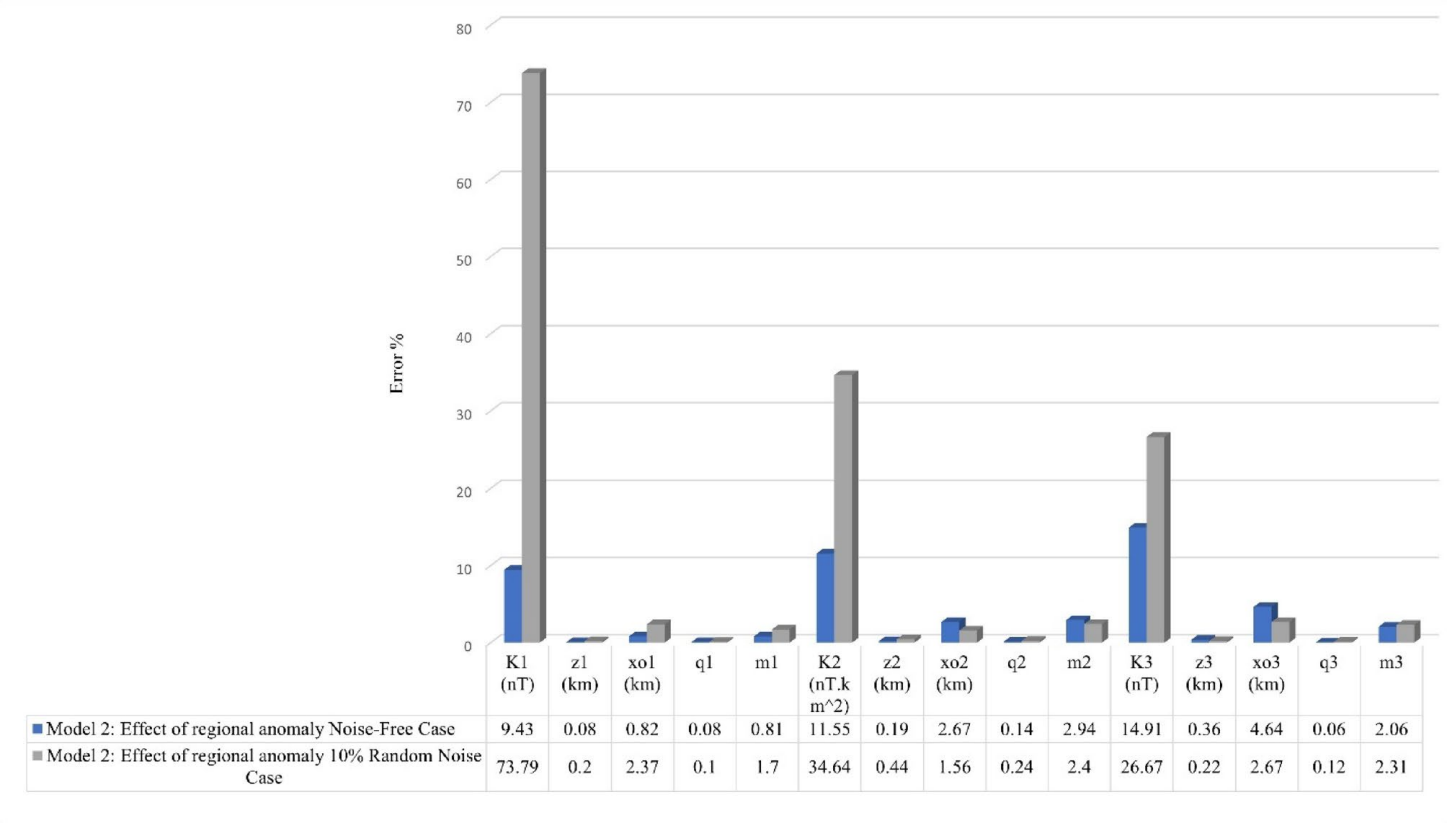
The relative error of each parameter illustrates the capability of our algorithm to accurately estimate the parameters and overseeing complex geological settings with the existing of neighbouring structures.

After introducing 10% random noise, the FHGBA was re-applied to estimate the parameters, yielding the following results: ($\:{K}_{1}$ = 2510 ± 73.79 nT, $\:{z}_{1}$ = 2.7 ± 0.2 km, $\:{q}_{1}$= 1 ± 0.1, $\:{x}_{o1}$ = −49.5 ± 2.37 km, and $\:{\theta}_{1}$ = −35˚ ± 1.7), ($\:{K}_{2}$ = 6470 ± 34.64 nT.km^2^, $\:{z}_{2}$ = 2 ± 0.44 km, $\:{q}_{2}$= 2 ± 0.24, $\:{x}_{o2}$ = 1 ± 1.56 km, and $\:{\theta}_{2}$ = −62˚ ± 2.04), and ($\:{K}_{3}$ = 3540 ± 26.67 nT, $\:{z}_{3}$ = 3.9 ± 0.22 km, $\:{q}_{3}$= 1 ± 0.12, $\:{x}_{o3}$ = 51 ± 2.67 km, and $\:{\theta}_{3}$ = 46˚ ± 2.31). The NRMSE for this scenario was 7.64 × 10^−6^ (Table 5), indicating a strong correlation between the computed and noisy anomalies (Fig. 8a), Fig. 8b shows FHG anomalies across various window values. Similar to the noise-free case, Fig. 8d demonstrates the iterative reduction of NRMSE, achieving convergence after approximately fifty-five iterations. Figure 8c complements this by illustrating the average NRMSE for each iteration, confirming steady progress toward the optimal solution. Figure 8e and f illustrate the variation in average loudness and emission rate as modelled through the bat algorithm. Figure 9 shows the relative errors to each parameter, which illustrates the capability of our algorithm to achieve accurately parameters estimate and overseeing complex geological settings with the existing of neighbouring structures.

**Table 5:** Estimation of subsurface parameters for a noisy synthetic magnetic field model. This table displays the results from the second synthetic example, incorporating a 10% level of noise while analyzing a thin sheet (dike), a horizontal cylinder, and an additional thin sheet (dike) alongside a third-order regional field represented by (0.0003 ($\:x$ -$\:\:{x}_{o}$)^3^+ 0.04 ($\:x$ -$\:\:{x}_{o}$) ^2^ + 0.15 ($\:x$ -$\:\:{x}_{o}$) − 200). It includes true model parameters, search ranges, estimated values for various window sizes of the fourth horizontal gradient (s = 1 to s = 10 km), and average parameter estimates with their uncertainties.

Model parameters	True model parameters	Search range	Estimated parameters										Avg. value ± Uncertainty	$\:{\varvec{U}}_{\varvec{O}\varvec{b}\varvec{j}}$
			s = 1 km	s = 2 km	s = 3 km	s = 4 km	s = 5 km	s = 6 km	s = 7 km	s = 8 km	s = 9 km	s = 10 km		
$\:{\varvec{K}}_{1}$(nT)	2500	2000–3500	2480	2470	2580	2590	2580	2400	2400	2480	2570	2550	2510 ± 73.79	7.64 × 10^−6^
$\:{\varvec{z}}_{1}$(km)	2.5	1–10	2.5	2.5	2.9	2.8	2.9	2.4	2.5	2.8	2.8	2.9	2.7 ± 0.2	
$\:{\varvec{x}}_{\varvec{o}1}$(km)	−50	−60 to −40	−47	−46	−51	−49	−49	−48	−48	−53	−52	−52	−49.5 ± 2.37	
$\:{\varvec{q}}_{1}$	1	0–2.5	0.9	0.9	0.85	1.05	1.15	1.1	1.1	0.95	0.95	1.05	1 ± 0.1	
$\:{\theta}_{1}$	−35	−90–90	−33	−34	−36	−35	−34	−34	−33	−36	−38	−37	−35 ± 1.7	
$\:{\varvec{K}}_{2}$(nT.km^2^)	6500	6000–7500	6450	6470	6480	6530	6530	6460	6440	6460	6450	6430	6470 ± 34.64	
$\:{\varvec{z}}_{2}$(km)	2	1–10	2.4	2.2	2.6	2.5	2.3	1.7	1.5	1.6	1.6	1.6	2 ± 0.44	
$\:{\varvec{x}}_{\varvec{o}2}$(km)	0	−10–10	−1	−1	0	1	1	1	3	3	0	3	1 ± 1.56	
$\:{\varvec{q}}_{2}$	2	0–2.5	1.7	1.8	2.1	2.2	2.3	2.1	2	1.8	1.7	2.3	2 ± 0.24	
$\:{\theta}_{2}$	−60	−90–90	−60	−64	−64	−61	−62	−59	−58	−63	−64	−65	−62 ± 2.4	
$\:{\varvec{K}}_{3}$(nT)	3500	3000–4500	3550	3500	3520	3570	3570	3530	3520	3580	3540	3520	3540 ± 26.67	
$\:{\varvec{z}}_{3}$(km)	4	1–10	3.7	3.9	3.9	3.7	3.6	4.2	4.2	4.1	4	3.7	3.9 ± 0.22	
$\:{\varvec{x}}_{\varvec{o}3}$(km)	50	−10–10	54	55	53	52	53	49	49	48	48	49	51 ± 2.67	
$\:{\varvec{q}}_{3}$	1	0–2.5	1.1	1.1	1.2	0.9	0.9	1	1.1	0.9	0.9	0.9	1 ± 0.12	
$\:{\theta}_{3}$	45	0–90	44	47	46	43	42	47	46	48	49	48	46 ± 2.31	

For establishing a baseline for comparison using the same parameters of the second model, the BA was applied directly to estimate the original parameters from the raw synthetic magnetic data. The recovering parameters are ($\:{K}_{1}$ = 2550 ± 35.36 nT, $\:{z}_{1}$ = 2.3 ± 0.14 km, $\:{q}_{1}$= 1.05 ± 0.04, $\:{x}_{o1}$ = −51 ± 0.71 km, and $\:{\theta}_{1}$ = −34˚ ± 0.71), ($\:{K}_{2}$ = 6450 ± 35.35 nT.km^2^, $\:{z}_{2}$ = 2.2 ± 0.14 km, $\:{q}_{2}$= 2 ± 0.00, $\:{x}_{o2}$ = 1 ± 0.70 km, and $\:{\theta}_{2}$ = −58˚ ± 1.41), and ($\:{K}_{3}$ = 3600 ± 70.72 nT, $\:{z}_{3}$ = 3.5 ± 0.35 km, $\:{q}_{3}$= 0.9 ± 0.071, $\:{x}_{o3}$ = 49 ± 0.71 km, and $\:{\theta}_{3}$ = 42˚ ± 2.12). The calculated NRMSE was 6.97.

After introducing 10% random noise, the BA was re-applied to estimate the parameters, yielding the following results: ($\:{K}_{1}$ = 2350 ± 106.07 nT, $\:{z}_{1}$ = 3.2 ± 0.49 km, $\:{q}_{1}$= 1.3 ± 0.21, $\:{x}_{o1}$ = −47± 2.12 km, and $\:{\theta}_{1}$ = −30˚ ± 3.5), ($\:{K}_{2}$ = 6620 ± 84.85 nT.km^2^, $\:{z}_{2}$ = 1.5 ± 0.35 km, $\:{q}_{2}$= 2.2 ± 0.14, $\:{x}_{o2}$ = −2 ± 1.41 km, and $\:{\theta}_{2}$ = −55˚ ± 3.54), and ($\:{K}_{3}$ = 3360 ± 98.99 nT, $\:{z}_{3}$ = 4.5 ± 0.35 km, $\:{q}_{3}$= 1.2 ± 0.14, $\:{x}_{o3}$ = 52 ± 1.41 km, and $\:{\theta}_{3}$ = 50˚ ± 3.53). The NRMSE for this scenario was 9.45.

The results further confirm the reliability of the FHGBA method, when the FHG anomalies are employed as input data for the algorithm. This procedure demonstrates superior performance and accurately recovering parameters under challenging and noisy conditions, compared to the results from the procedure of using the raw magnetic data directly as input to the BA. These test results highlight the FHGBA approach’s potential as a valuable tool for interpreting magnetic data, which frequently involves substantial uncertainties in real-world scenarios.

### Real datasets: the Faro magnetic anomaly, yukon, Canada

**Fig. 10:**
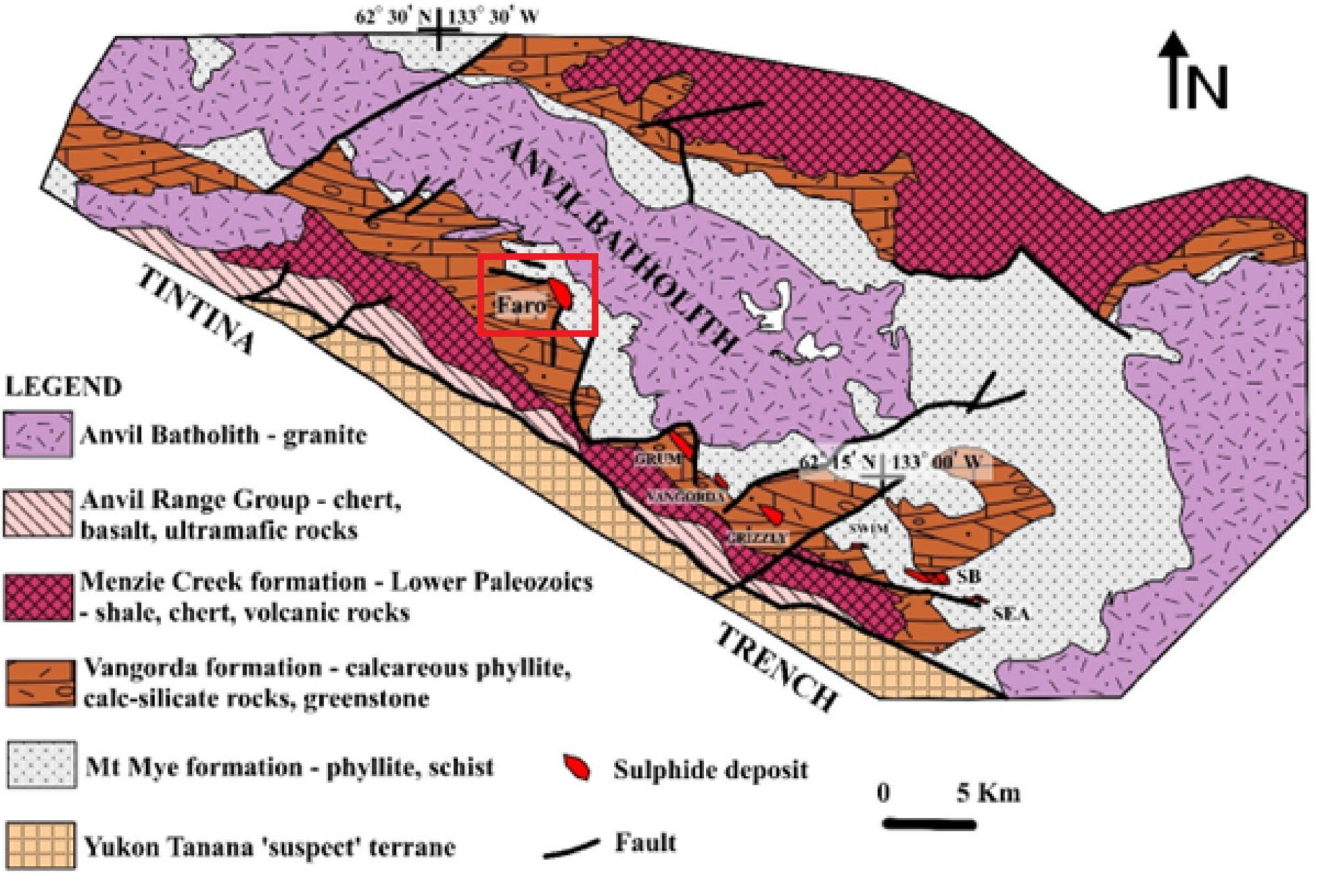
Geological overview of the Anvil District in Canada, including the Faro Sulphide Deposit^66^. The study area is outlined in solid red rectangular.

**Fig. 11:**
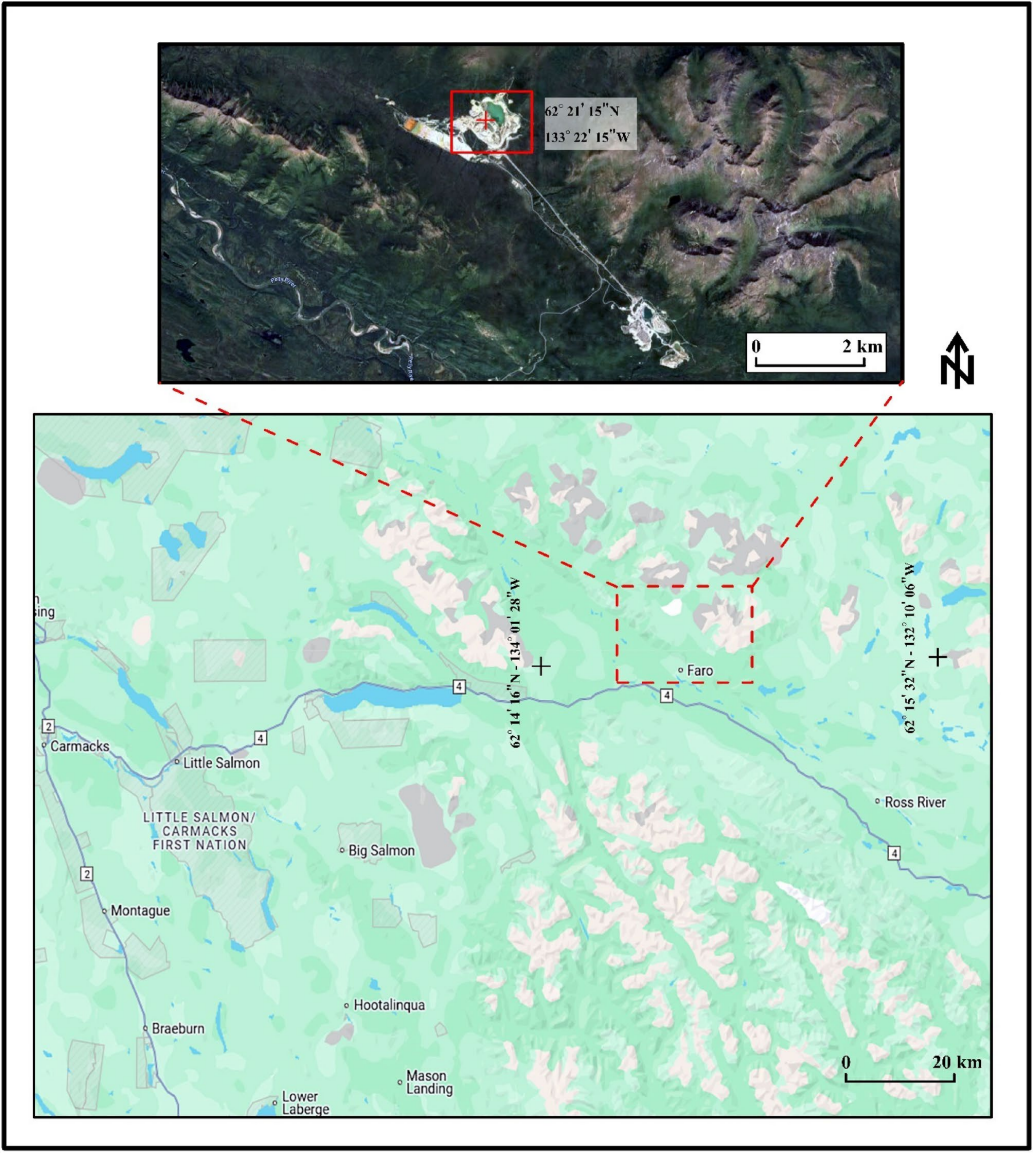
Location Map of the Faro Mine Complex, Canada. Using different layers of google maps 2025, the dashed red rectangle refers to the location of the study area, the solid red rectangle refers to detailed location of the site, and the red plus sign is the location of the profile.

The Faro Mine located approximately 15 km north of the town of Faro in south-central Yukon, Canada^66^, lies within a region shaped by the Yukon Plateau and the nearby Anvil Range Mountains, with elevations surpassing 1800 m. This mine is positioned in the northwest portion of the Faro Complex (Fig. 10), where the geological framework is strongly influenced by the characteristics of the Anvil Range (Fig. 11).

**Fig. 12:**
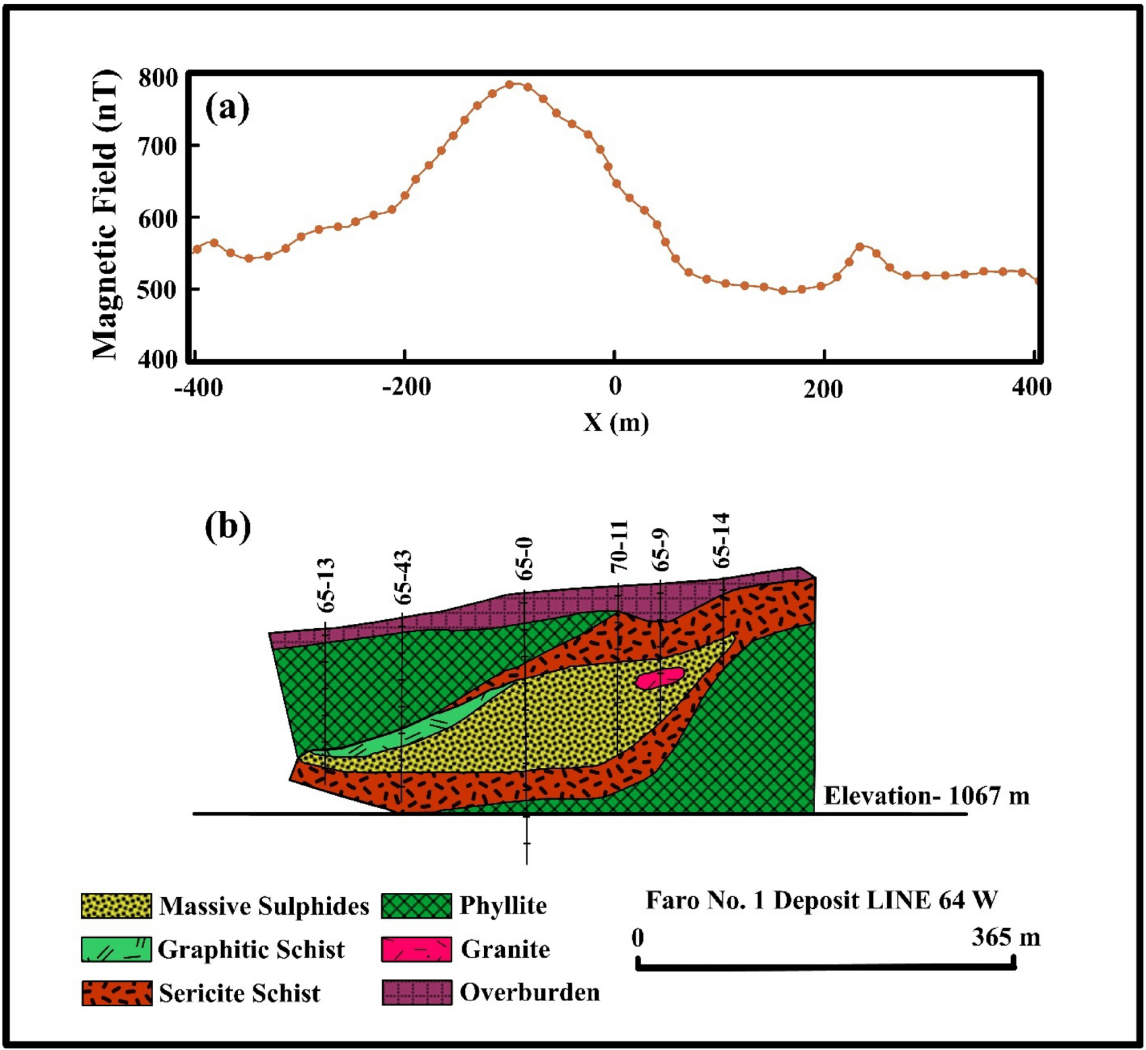
Magnetic anomaly and geological cross-section of the Faro Lead–Zinc Deposit, Yukon, Canada. **a** Magnetic field profile. **b** Subsurface geological model with boreholes^68^.

A geophysical survey of the Faro district incorporated magnetic and gravity methods to explore subsurface ore deposits. Magnetic measurements over the Faro deposit (Fig. 12a) identified a shallow-gradient anomaly^67^, attributed to a lead-zinc sulfide ore body situated roughly 100 m beneath the contact between the Mount Mye and Vangorda formations (Fig. 12b). The Mount Mye Formation has undergone alteration into biotite-muscovite schist, while the Vangorda Formation has metamorphosed into thick, banded calcsilicate rocks^68^.

**Fig. 13:**
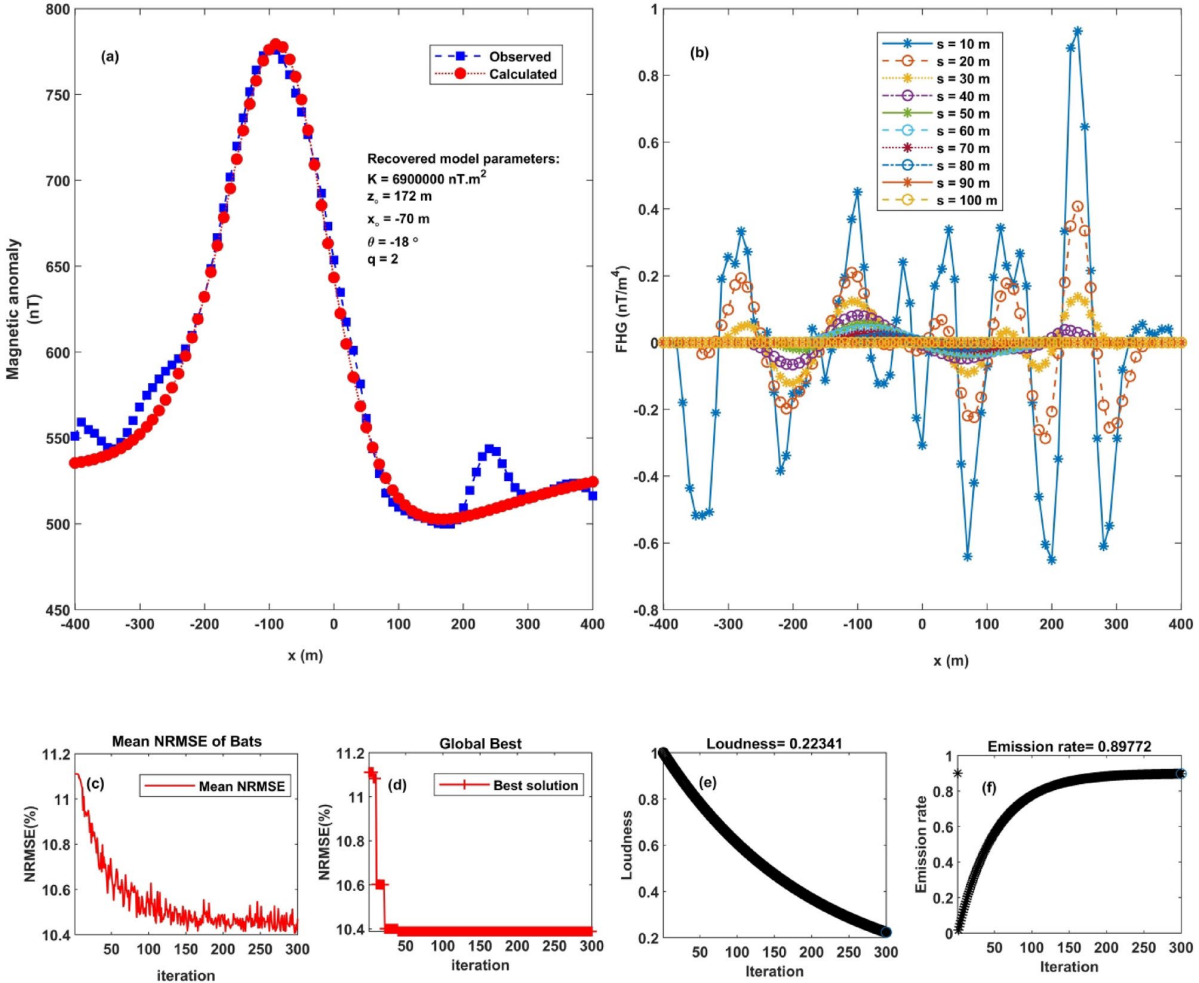
Application of FHGBA on Faro Lead-Zinc Deposit. **a** Observed magnetic anomaly and computed response using FHGBA. **b** FHG anomalies for varying ‘s’ values. **c** Average NRMSE of bats. **d** NRMSE of global optimum ($\:{\varvec{U}}_{\varvec{O}\varvec{b}\varvec{j}}$) relative to iterations, **e** Loudness variation. **f** Emission rate of bats.

Initially, Brock^69^ estimated the deposit’s mass to be 44 million tons, based on assumed densities of 3.65 g/cm³ for the ore and 2.75 g/cm³ for the host rock. Subsequent drilling results confirmed this estimate, with a revised mass of approximately 46 million tons^68–70^. A magnetic profile spanning 801 m over the “Faro No. 1 Deposit” (Fig. 13a) was digitized at 10-meter interval. Analysis of the magnetic anomaly using the FHGBA method facilitated the determination of critical source parameters (Fig. 13a and b).

Figures 13c and d depict the normalized root-mean-square error (NRMSE) for both the best global solution ($\:{U}_{Obj}$) and the average NRMSE across all iterations. Figure 13e and f illustrate the variation in average loudness and emission rate, as modelled through the bat algorithm in response to the magnetic anomaly.

The optimal parameters of the anomaly source, determined at a minimum $\:{U}_{Obj}\:$value of 10.6 were: K = 6,900,000 ± 81,650 nT.m^2^, z = 172 ± 4.42 m, $\:{x}_{o}$= −70 ± 2.67 m, θ = −18° ± 1.7 and q = 2 ± 0.21 (Table 6). These results suggest that the anomaly originates from a horizontally oriented cylindrical body. Figure 13a illustrates the excellent agreement between the observed and modeled magnetic anomalies over the Faro deposit.

**Table 6:** Results of parameter Estimation for the Faro magnetic anomaly, yukon, canada. This table presents the outcomes of applying our algorithm to a real field magnetic data profile from the Faro magnetic anomaly. It includes true model parameters, search ranges, estimated values, and associated uncertainties, highlighting the effectiveness of the algorithm in analyzing the magnetic characteristics of this geological feature.

Model parameters	Search range	Estimated parameters										Avg. value ± Uncertainty	$\:{\varvec{U}}_{\varvec{O}\varvec{b}\varvec{j}}$
		s = 1 m	s = 2 m	s = 3 m	s = 4 m	s = 5 m	s = 6 m	s = 7 m	s = 8 m	s = 9 m	s = 10 m		
K (nT)	68 × 10^5^ – 71 × 10^5^	7,000,000	7,000,000	6,900,000	7,000,000	6,900,000	6,900,000	6,800,000	6,900,000	6,800,000	6,800,000	69 × 10^5^ ± 81,649	1.06 × 10
z (km)	10–200	176	170	164	172	174	176	168	168	174	178	172 ± 4.42	
$\:{\varvec{x}}_{\varvec{o}}$(km)	−100–100	−72	−66	−68	−67	−68	−72	−72	−69	−73	−73	−70 ± 2.67	
q	0–2.5	1.8	1.8	2.2	2.1	2.1	2.3	1.9	1.9	1.7	2.2	2 ± 0.21	
$\:\theta$	−90–90	−16	−19	−17	−17	−19	−18	−21	−20	−17	−16	−18 ± 1.7	

Gravity anomalies detected during the survey were interpreted using the R-parameter imaging technique^71^, which estimated the depth to the center of the anomaly to be approximately 195 m. This interpretation also suggested a horizontally oriented cylindrical shape. The FHGBA method provided a depth estimate of 172 m, closely aligning with geological and drill-hole data (Fig. 12b). This consistency with both geological findings and previous gravity analyses underscores the reliability of the FHGBA method in accurately characterizing Faro anomaly.

## Conclusions

The FHGBA has proven to be a robust and effective tool for magnetic data inversion. Through synthetic and real-world applications, the method demonstrated its capability to accurately estimate subsurface parameters under various challenging conditions, including the presence of noise and the influence of regional anomalies. The FHGBA accurately recovered original parameters across multiple test cases, with minimal deviation, even when synthetic data was subjected to 10% noise levels or influenced by complex regional effects. The algorithm efficiently minimized the NRMSE, consistently converging to optimal solutions in a limited number of iterations. Applied to magnetic data from the Faro Mine in Yukon, Canada, the FHGBA produced parameter estimates that were highly consistent with geological expectations and corroborated by previous geophysical surveys and drilling data. The estimated depth and source characteristics aligned closely with independent analyses, confirming the algorithm’s practical utility. The integration of the FHG with the BA provided enhanced resolution and noise suppression, enabling reliable subsurface imaging. These findings underscore the FHGBA’s potential as a valuable tool for geophysical exploration.

## Data Availability

The datasets used and/or analyzed during the current study available from the corresponding author on reasonable request.
